# Improvement of Magnetic Particle Hyperthermia: Healthy Tissues Sparing by Reduction in Eddy Currents

**DOI:** 10.3390/nano11020556

**Published:** 2021-02-23

**Authors:** Alexandros Balousis, Nikolaos Maniotis, Theodoros Samaras

**Affiliations:** 1 Department of Physics, Aristotle University of Thessaloniki, 54124 Thessaloniki, Greece; ampalous@physics.auth.gr (A.B.); theosama@auth.gr (T.S.); 2 Department of Physics, University of Malta, 2080 Msida, Malta

**Keywords:** magnetic particle hyperthermia, magnetic nanoparticles, eddy currents reduction, tissue sparing

## Abstract

Attenuation of the unwanted heating of normal tissues due to eddy currents presents a major challenge in magnetic particle hyperthermia for cancer treatment. Eddy currents are a direct consequence of the applied alternating magnetic field, which is used to excite the nanoparticles in the tumor and have been shown to limit treatment efficacy in clinical trials. To overcome these challenges, this paper presents simple, clinically applicable, numerical approaches which reduce the temperature increase due to eddy currents in normal tissue and simultaneously retain magnetic nanoparticles heating efficiency within the tumor. More specifically, two protocols are examined which involve moving the heating source, an electromagnetic coil, relative to a tumor-bearing phantom tissue during the exposure. In the first protocol, the linear motion of the coil on one side with respect to the hypothesized tumor location inside the phantom is simulated. The estimated maximum temperature increase in the healthy tissue and tumor is reduced by 12% and 9%, respectively, compared to a non-moving coil, which is the control protocol. The second technique involves a symmetrical variation of the first one, where the coil is moving left and right of the phantom in a bidirectional fashion. This protocol is considered as the optimum one, since the estimated maximum temperature rise of the healthy tissue and tumor is reduced by 25% and 1%, respectively, compared to the control protocol. Thus, the advantages of a linearly moving coil are assessed through tissue sparing, rendering this technique suitable for magnetic particle hyperthermia treatment.

## 1. Introduction

Magnetic particle hyperthermia (MPH) is a promising technique for cancer treatment [1,2,3,4]. The method involves delivering magnetic nanoparticles (MNPs), suspended in an aqueous solution also known as ‘ferrofluid’, into tumors either through direct injection or via passive or active targeting following intravenous administration [5,6]. Once accumulated in the tumor area, MNPs are exposed to an external alternating magnetic field (AMF) that causes reversal of their magnetic moments, activating mechanisms of energy deposition in the form of heat. The basic heating mechanism is attributed to Néel and Brownian relaxation mechanisms [7]. Rosensweig [8] developed a model that calculates the MNPs’ volumetric power dissipation in a ferrofluid, by taking into account both of the above relaxation theories, through analytical relationships. Fundamental advantages of MPH over alternative hyperthermia modalities include power dissipation only through magnetic nanoparticles, providing better localization and leaving healthy tissues intact [4,9], while modifications in MNPs morphology enable adjustments of magnetic properties and control overheating capabilities [10,11]. In addition, the penetration depth of the AMF exceeds that of alternative electromagnetic or ultrasound stimulus [12]. As a result, MPH is more suitable for reaching deep-seated tumors [13].

Despite its impressive advantages, MPH is slowly adopted in clinical practice due to several unsolved challenges and limitations. One of them is that the externally applied AMF induces eddy currents inside the human body, which result in unwanted heating of normal tissues [14]. The magnitude of eddy current depends, in part, on the AMF characteristics and the geometry of the body part exposed to it [15,16]. Quantification of allowable AMF conditions that prevent discomfort, damage and pain due to excessive eddy currents, is an obligatory task. To date, the only reliable tolerance limit introduced by Atkinson et al. [17] sets an upper value for the product H_0_∙f = 4.85 × 108 A/(m∙s), also known as the ‘Brezovich limit’, where H0 and f are the AMF amplitude and frequency, respectively. The efficiency-limiting nature of eddy current heating (ECH) was observed in a recent clinical trial, where patient discomfort was reported [18,19].

Until now, much of the literature regarding increasing the efficacy of MNP hyperthermia therapy has focused on increasing MNPs’ specific absorption rate (SAR) [20,21,22] or increasing the concentration of MNPs in the tumor [23,24,25]. There are only a few studies focused on decreasing the maximum temperature resulting from ECH in order to increase therapeutic ratio. Stigliano et al. [26] presented a coil technique for moving MNPs containing tissue phantom, in order to manipulate the tissue exposed to the field and also to decrease thermal dose due to ECH, by considering the placement of tissue in time and space, relative to the AMF, or vice versa. Although the decrease in the maximum heat deposition in non-cancerous regions is achieved by the authors, there are some limiting factors concerning the implementation of this technique. The first one is the erosion of the sample phantom, which is often required to be replaced in experiments. The second one is the complexity of the Pennes bioheat equation [27] that needs to be solved for the computational evaluation of treatment protocols. In order to overcome this complexity, Neufeld et al. [28] developed an approximation approach to predict the temperature increase during magnetic resonance imaging (MRI) radiofrequency exposure. The approximation is based on the assumption that the temperature increase exhibits exponential behavior and eventually tends to equilibrium.

In this work, we present a simple and clinically applicable technique for decreasing the maximum temperature induced by ECH inside healthy tissues. We improve the coil movement technique proposed by Stigliano et al. by using the heat transfer approximation of Neufeld et al. The first case study explores the effects of a linear motion of the coil on one side with respect to the hypothesized tumor location inside the phantom, with a brief downtime of the AMF. The second case study involves a symmetrical linear motion, bidirectional with respect to the assumed tumor location, without downtime. Our work aims to demonstrate the conditions for enhanced healthy tissue sparing without compromising the MPH treatment efficiency.

## 2. Materials and Methods

### 2.1. Heating Mechanisms during MPH

There are two main mechanisms concerning tissue heating during MPH treatment. The first one is the MNPs’ power dissipation in the tissue, denoted as ***SAR_MNP_***, and quantified by Rosensweig’s theoretical model [8] which takes into account the Néel and Brownian relaxation times. The MNPs’ volumetric power dissipation ***P*** is given in Equation (1),

(1) $$P=\pi \mu_{0}\chi_{0}H_{0}^{2}f\frac{2\pi f\tau }{1\;+\;{\left(2\pi f\tau \right)}^{2}}$$

where $\chi_{0}$ is the MNPs’ magnetic susceptibility and τ is the total relaxation time. In order to estimate the specific absorption rate, ***SAR_MNP_***, we use Equation (2),

(2) $$SAR_{MNP}=\frac{P\;\times \;\varphi }{\rho_{MNP}}$$

where ***φ*** is the MNPs’ volume fraction and ***ρ_MNP_*** is the MNPs’ density. The second heating source is the heating due to electric currents induced by the AMF within a conductive medium, such as tissue. This heating source is represented by the specific absorption rate ***SAR_EC_*** and calculated using Equation (3),

(3) $$SAR_{EC}=\frac{1}{2\rho_{t}}\sigma {\left|E\right|}^{2}=\frac{1}{2\rho_{t}}\sigma {\left(\pi \mu_{0}\left|H\right|fr\right)}^{2}$$

where ***f*** is the frequency of the AMF, ***ρ_t_*** is the density of tissue, ***σ*** is the tissue conductivity, ***r*** is the radial position in tissue from the center of the coil, $\left|E\right|$ is the electric field and $\left|H\right|$ is the magnetic field amplitude. The relative magnetic permeability of tissue is taken as ***μ_r_*** = 1. The total ***SAR*** in the tissue is evaluated by Equation (4),

(4) $$SAR=SAR_{EC}+SAR_{MNP}$$

### 2.2. Model Description

#### 2.2.1. Geometry

**Figure 1:**
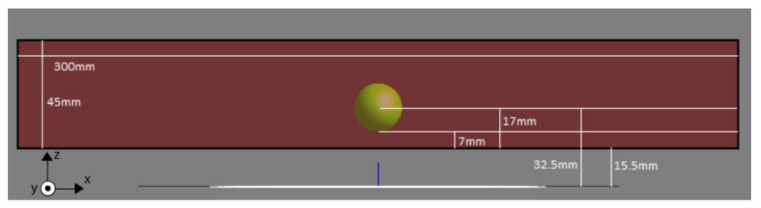
Phantom dimensions and tumor positioning (yellow circular area) at the center of the phantom.

In order to investigate the possibility of improving the treatment using a moving source, simulations were performed for a rectilinear tissue phantom, simulating a part of the patient’s body. The numerical phantom dimensions were set at 300 mm × 300 mm × 45 mm. Within the phantom, we assumed a spherical tumor of 10 mm radius, with its center 17 mm above the bottom surface of the phantom as shown in Figure 1. The tumor was not assigned different physical properties from healthy tissue; its presence in the model denotes the treatment target volume, where MNPs were supposed to have accumulated. The coil generating the AMF was located 15.5 mm below the phantom (Figure 1). It was a concentric 10-turn circular coil, with an inner diameter of 40 mm and outer diameter of 70 mm.

**Figure 2:**
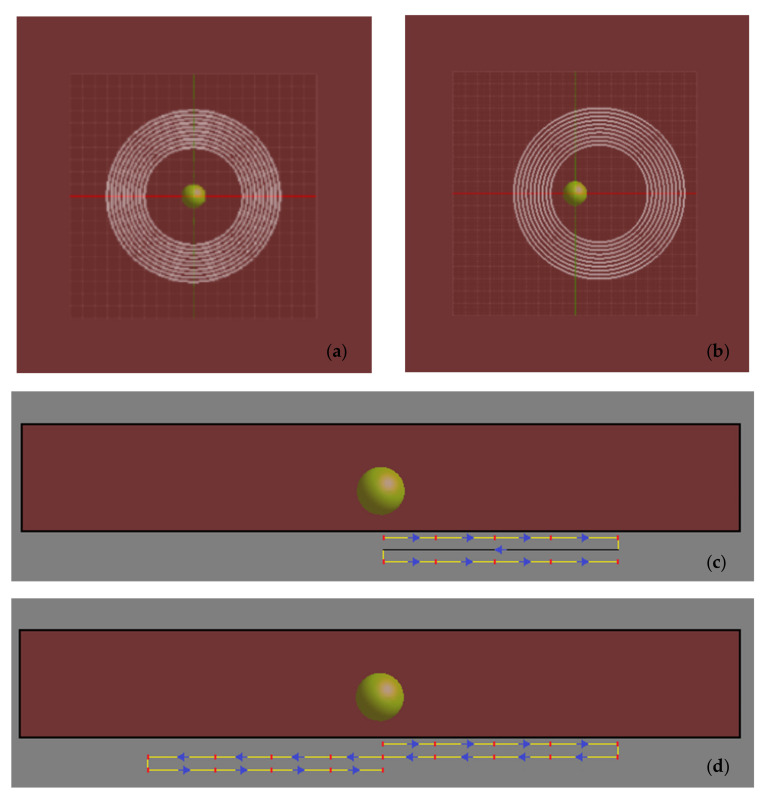
(**a**) Initial position of the coil. (**b**) Final position of the coil during its linear motion along x- axis (center of coil at 20 mm away from its initial position). (**c**) Cross-sectional image of the simulated scheme for the unidirectional case with the coil trajectory depicted below the phantom. The initial position of the coil center coincided with the phantom center and is defined as x = 0. The blue arrows depict the direction of coil motion. Each red point corresponds to the position where the coil temporarily remains while the moving step is depicted with a yellow line and equals 5 mm (distance from point to point). The black line shows the return of the coil to the initial position with a field downtime of 2 s. (**d**) Cross-sectional image of the simulated scheme for the bidirectional case. After the coil reaches its maximum displacement at x = 20 mm, it turns to the opposite direction until it reaches the position x = −20 mm, with respect to the phantom center, without the field downtime.

A top view of the simulated scheme for the first protocol of movement studied, is depicted in Figure 2a,b. This protocol involved the coil moving in 5 mm steps in the x-axis direction, with a 20 mm end position. Initially, the coil center was aligned to the tumor center (Figure 2a). The coil moved to the next position (+5 mm along the x axis) and remained there for some time, then it moved again, and the process was repeated until it reached its final position (Figure 2b). The coil current (i.e., the external AMF) was then switched off and the coil was returned to its original position after a total time of 2 s. The above procedure was repeated periodically until the end of treatment. This case is referred to as ‘unidirectional’ in the following. To better clarify this protocol of coil movement, a cross‐sectional view of the simulated scheme is illustrated in Figure 2c.

The second case differed from the first in the range of movement of the coil. This time the coil was allowed to move again along the x-axis, but symmetrically with respect to its initial position, between the positions of x_min_ = −20 mm and x_max_ = +20 mm and without the field downtime of 2 s. This protocol of coil movement is shown in the phantom cross-sectional image of Figure 2d. The starting position remained the center of the phantom, and this case will be referred to as ‘bidirectional’. For both cases, the control (or reference) case corresponded to a fixed coil without downtime (Figure 2a).

#### 2.2.2. Properties and Conditions

The phantom and the tumor had the same physical properties: ***ρ_t_*** = 998 kg/m^3^, ***σ*** = 0.6 S/m. The MNPs were assumed as 10 nm in diameter, made of magnetite, with a density of ***ρ_MNP_*** = 5240 kg / m^3^ and $\chi_{0}$ = 22.13. This size results in a pure superparamagnetic relaxation behavior for magnetite MNPs [10] and their heating losses can be well described by Equation (1). In addition, the blocking temperature ***T_B_***, above which typical characteristics of superparamagnetic characteristics are observed (thermally unstable nanoparticles), was taken from experimental data of our previous work on magnetite MNPs of 10 nm [29] and was equal to 160 K, a value lower than the working temperature (***T*** = 300 K). Then, the effective anisotropy ***K_eff_*** of MNPs is extracted by using the relationship ***K_eff_*** = 25***k_B_T_B_/V*** = 10.55 × 10^5^ erg/cm^3^, where ***k_B_*** is the Boltzmann constant and ***V*** is the MNPs volume. Since MNPs were considered to be stabilized within the tumor, it was assumed that the dominant mechanism of energy deposition was via Néel relaxation with [3] ***τ_N_*** = $\frac{\tau_{0}}{2}\sqrt{\frac{\pi k_{B}T}{K_{eff}V}}exp\left(\frac{K_{eff}V}{k_{B}T}\right)$ = 1.34 × 10^−6^ s, where *τ*_0_ is taken as being equal to 10^−10^ s. The MNPs’ concentration in the solution was taken as ***C*** = 4 mg/mL, which corresponded to a volume fraction ***φ*** = ***C/ρ_MNP_*** = 0.07%, a value typically used in in vivo MPH [30,31,32].

The AC current frequency of the coil was set to 162 kHz. The total treatment lasted for 3600 s. The value of 37 °C was considered as the body temperature for analyzing the conclusions. Thermoregulation was not considered in the calculations.

### 2.3. Numerical Electromagnetic Calculations

In order to estimate the magnetic and induced electric fields produced by the coil, we used the low-frequency electromagnetic field simulation module of Sim4Life (ZMT, Zurich, Switzerland), which solves the time harmonic Maxwell equations by implementing a quasi-static approximation. The rectilinear grid used as the computational space for the solution included about 3 million cells (voxels), with increased accuracy close to the target volume area (0.5 mm resolution).

### 2.4. Our Algorithm

**Figure 3:**
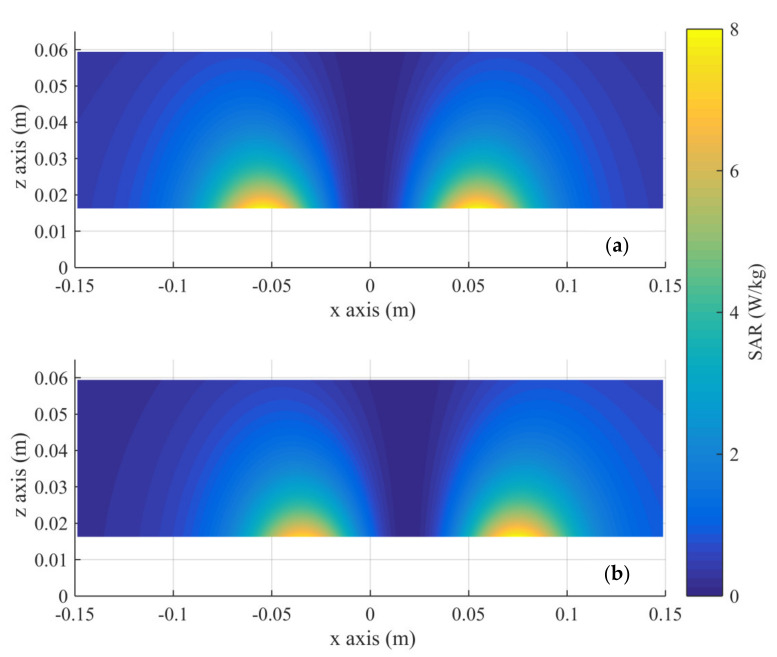
$SAR_{EC}$ distribution in the tissue simulant on an x-z slice at the phantom’s center. The coil is located at z = 0 and (**a**) at the initial position (x = 0) and (**b**) at the maximum displacement.

The purpose of the algorithm used in this study was to find a treatment protocol that would result in a hyperthermic temperature inside the tumor volume (target volume) but would tolerate temperature rise anywhere else (in the healthy tissue), due to eddy currents. The ‘protocol’ is the temporal configuration of the coil’s motion, i.e., the amount of time it remains at the various positions. The algorithm includes the following steps:

1. The volume data resulting from the numerical solution of the electromagnetic problem are imported. The $SAR_{EC}$ is maximum on the surface of the tissue phantom just above the turns of the coil (Figure 3a). Therefore, this is the ‘hotspot’ of the eddy current heating. When the coil is moved along the x-axis, the position of the ‘hotspot’ changes (Figure 3b). During the implementation of any protocol, the positions of the ‘hotspots’ and the temperature in them due to the eddy currents, are monitored with time.
2. At each coil position, the temperature increase over time at the ‘hotspot’ and at the center of the tumor (target) volume is calculated with Equation (5), using the approximation of Neufeld et al.,
  (5) $$\Delta {Τ}_{i+1}=\Delta T_{i}+\left(1-e^{-\Delta t/\tau }\right)\left(cSAR_{i}-\Delta T_{i}\right)$$
  where $\Delta {Τ}_{i}$ is the temperature increase at a specific time ***t_i_***, $\Delta {Τ}_{i+1}$ is the temperature difference at the next time step, Δt is the time step,$\;SAR_{i}$ is the maximum SAR value at the calculation position at time ***t_i_***, and ***c*** and ***τ*** are constants related to electromagnetic heating of the tissue phantom; ***τ*** = 580 s and ***c*** = 0.375 K/(W/kg) [28]. The time step is set to **∆*t*** = 0.1 s. The value of $SAR_{i}$, at the center of the tumor or the ‘hotspot’ identified at each step, is calculated from Equation (4), during the course of the coil’s motion. We assume that MNPs are located only inside the tumor volume and not in the healthy tissue.
3. In order to assess various protocols, time intervals are assigned to each coil position. The values of these intervals range between 5 and 50 s (with a step of 5) and they correspond to the time the coil will remain at each position before moving to the next one. More than 10^5^ protocols are examined, and the temporal temperature evolution in the tumor center and in the ‘hotspots’ is calculated for them.
4. Since the aim of the algorithm is to minimize healthy tissue heating due to eddy currents, the protocol that leads to the lowest temperature rise at the end of the treatment is chosen, both for the unidirectional and bidirectional case. The temperature rise at the tumor center is then compared with the control case (fixed coil) to note any lowering of the final treatment temperature.

## 3. Results

**Figure 4:**
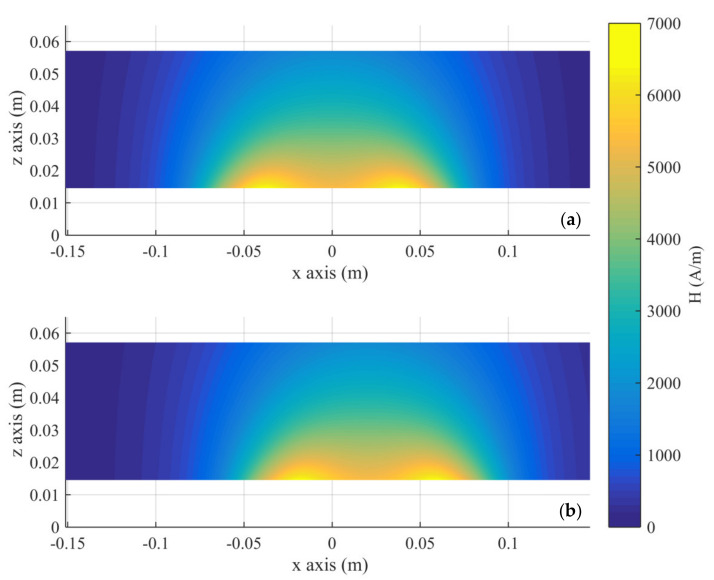
Magnetic field distribution in the tissue simulant on an x-z slice at the phantom’s center. The coil is located at z = 0 and (**a**) at the initial position (x = 0) and (**b**) at the maximum displacement.

The $SAR_{EC}$ and magnetic field distributions in healthy tissue resulting from the numerical solution of the electromagnetic problem are presented in Figure 3 and Figure 4, respectively. The plane depicted is the mid-plane of the phantom, including a diameter of the coil.

The protocols that generated the lowest final maximum temperatures in healthy tissue for each case study were the following: In the unidirectional case the coil remained for 5 s at each position. In the bidirectional case, the coil remained again for 5 s at each position, with the exception of the maximum displacement (±20 mm), where the coil remained for 30 s.

**Figure 5:**
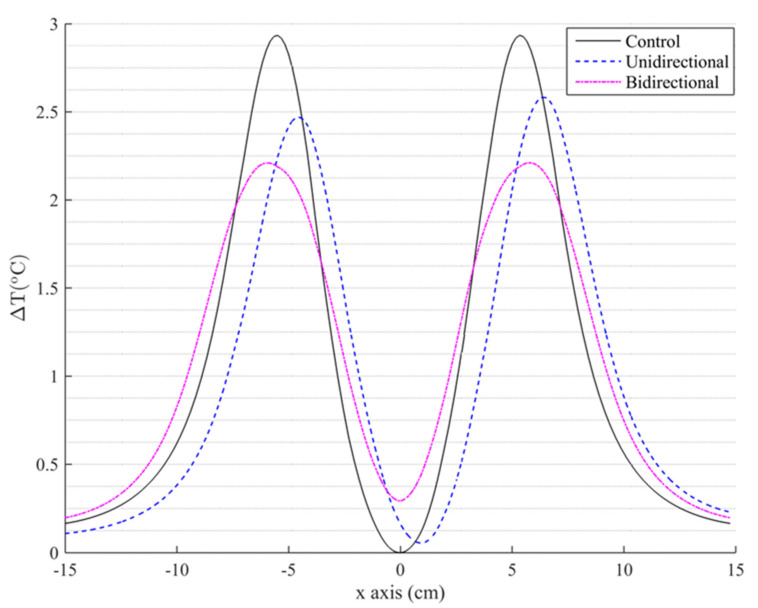
Spatial distribution of the highest temperature increase during the treatment, along the x-axis and at the bottom of the phantom tissue for control and both motion study cases.

The distribution of temperature rise at the end of the MPH treatment along the x-axis at z = 0.015 m, which corresponds to the bottom of the phantom, is presented in Figure 5.

Both protocols presented a notable decrease in the maximum temperature of healthy regions compared to the control case, in which ΔΤ_max_ = 2.9 °C. The unidirectional protocol led to a reduction in ΔΤ_max_ by approximately 12%, while the bidirectional protocol managed to achieve a drop of approximately 25%. The significant reduction in hotspot temperature was accompanied by a slight increase in temperature in regions which previously exhibited less heating. This heating, however, was negligible compared to the control case. Both the decrease in ΔΤ in some regions and the increase in some others are a result of the spreading of the cumulative energy delivered by eddy currents to a wider area.

**Figure 6:**
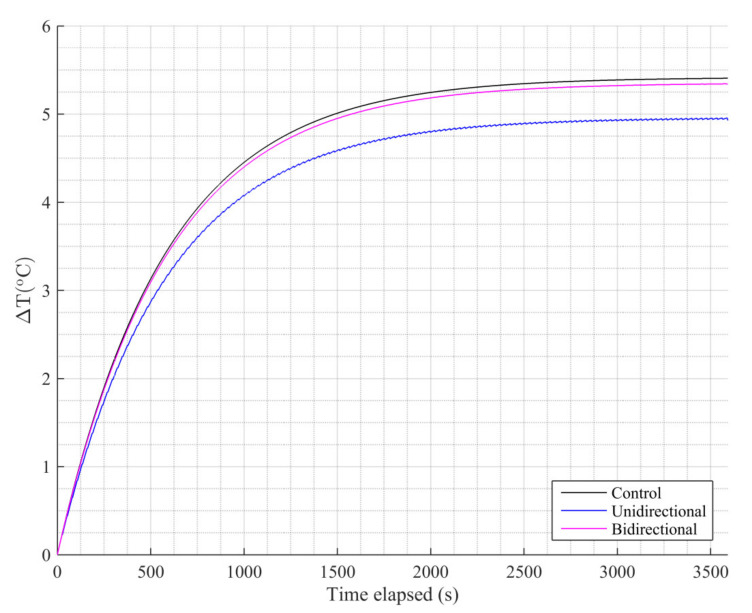
Temperature increase at the center of the tumor (target) volume during treatment.

Next, we estimated the impact of the coil motion on the temperature increase in the tumor, as illustrated in Figure 6. The tumor’s final temperature in the control case was approximately 5.4 °C.

The MNPs’ heating efficiency was reduced when applying either protocol, because the magnetic field they were exposed to was not constantly the maximum possible (on the axis of the coil). As a consequence, the tumor’s maximum temperature reduced by 9% for the unidirectional case and 1% for the bidirectional one. This result, in combination with the 25% reduction in temperature increase in healthy tissue compared to the control case, allows us to characterize the bidirectional case as the optimal protocol applied.

## 4. Conclusions

The aim of this work was to assess the potential benefits of a linearly moving coil for the improvement of MPH treatments via enhanced tissue sparing, while simultaneously exploring alternative methods for a more computationally efficient simulation of treatment protocols. Existing works have focused on methods of increasing MNP heating in tumors without attempting to modify eddy currents heating. We have succeeded in developing a protocol for the coil’s motion that significantly reduced the temperature rise in healthy tissue (25%) while minimally affecting the maximum temperature rise in the treatment target (1%). In clinical application of MPH, the result of heating may be gradual apoptosis or even necrosis of cells that are subjected to heat shock within the hyperthermia window of 42−45 °C. Consequently, the bidirectional protocol was able to sustain the desired temperature increase inside the tumor region (within 5−8 °C above an assumed body temperature of 37 °C), while in the healthy tissue region the temperature variation remained below the critical temperature rise of 5 °C, illustrating eddy current mitigation, which is a prerequisite for subsequent clinical application.

The presented methodology can also be adapted to deal with other medical electromagnetic applications, or to any other diagnostic or treatment modality involving temporally and spatially changing local energy deposition. In the near future, an experimental validation of the methodology is planned. After that, it will be possible to perform an extensive parametric study of different sizes and concentrations of magnetite nanoparticles, different alternate current frequencies of the coil, and various cancer site positions in realistic numerical human models.

## Data Availability

The data presented in this study are available in this article.
